# “And then you start to loose it because you think about Nutella”: The significance of food for people with inflammatory bowel disease - a qualitative study

**DOI:** 10.1186/s12876-015-0322-2

**Published:** 2015-07-30

**Authors:** Alexander Palant, Janka Koschack, Simone Rassmann, Gabriele Lucius-Hoene, Michael Karaus, Wolfgang Himmel

**Affiliations:** 1 Department for General Practice, University Medical Center Göttingen, Humboldtallee 38, 37073, Göttingen, Germany; 2 Institute of Psychology, University of Freiburg, Freiburg, Germany; 3 Evangelic Hospital Göttingen-Weende, Göttingen, Germany

**Keywords:** Inflammatory bowel disease, Diet, Narration, Qualitative research

## Abstract

**Background:**

Many patients with inflammatory bowel disease strongly believe that food or certain food products heavily influence the symptoms or even trigger acute flare-ups. Unfortunately, there is no generalizable information for these patients, and therefore no effective diet has been identified to date.

**Methods:**

The narrative interviews we used for this study provide the basis for the German website www.krankheitserfahrungen.de. Maximum-variation sampling was used to include a broad range of experiences and a variety of different factors that might influence people’s experiences. The sample included men and women of different age groups and social and ethnic backgrounds from across Germany. The interviews were analyzed using grounded theory.

**Results:**

Four interrelated categories emerged: managing uncertainty, eating: between craving and aversion, being different and professional help as a further source of uncertainty. The most important issue for our responders was the handling of uncertainty and to find a way between desire for, and aversion against, eating. Many participants described difficulties during formal social occasions such as weddings, birthdays, or when going out to a restaurant.

**Conclusions:**

Many of the experiences the participants reported in their daily struggle with food and their illness, such as cravings for and abstaining from certain foods, were rather unusual and often stressful. Because they decided not to go out in public any longer, some of the interviewees experienced even more social isolation than they did before. Health professionals need to become more involved and not only advice about food and eating, but also help their patients find strategies for avoiding social isolation.

## Background

Inflammatory bowel diseases (IBD) such as ulcerative colitis (UC) and Crohn’s disease (CD) are chronic relapsing diseases of the gastrointestinal tract and currently cannot be cured [1]. The most common symptoms of IBD are bowel obstruction, diarrhea with passage of blood, abdominal pain, and weight loss [2]. The incidences are increasing worldwide. The prevalence of IBD is approximately 200 cases per 100,000 inhabitants in the Western world, with 2.4 million patients in Europe and 1.3 million in the US [3].

The cause of IBD is still unknown but thought to be multifactorial [2]. Environmental factors play an important role, with diet being a prominent candidate [4]. Diet is also often the first behavioral factor that is manipulated by individuals with an IBD after the onset of the symptoms [5]. The idea that there might be a connection between IBD and food has first been discussed about 70 years ago [6], but it is a very complex matter and still not fully understood. In a review of lay literature and different internet sources, Hou et al. [7] discussed patient-targeted dietary recommendations. They concluded that there is no generalizable information for IBD patients and therefore no effective diet identified so far. While food intolerances are very frequent among IBD patients, individuals react differently to exposure or exclusion from various foods, so that no general dietary recommendations can be made from a professional point of view [8, 9].

The validity of research results about particular food or food groups and their influence on the course of IBD, for example, sugar, milk, eggs, fruit, vegetables, protein, and fat [8, 10, 11], is limited because of its retrospective character. It is very difficult if not impossible to recall dietary intake prior to developing the illness, so that a current diet might be influenced by illness-induced changes [4]. Consequently, many conflicting results have been reported [7]. However, many patients with IBD strongly believe that food or certain food products heavily influence the symptoms or even trigger acute flare-ups [9, 12, 13]. It is all the more surprising that patients’ perception of food and how it influences their eating behavior and related social activities has not been studied in more detail [13, 14], although approximately 90 % of the patients in another study changed their diets after the diagnosis [5].

The need for information is another important issue for patients with IBD because quality of life negatively correlates with a patient’s perceived information level [15]. In a recent study, 80 % of the patients rated dietary guidance as important, including “diet changes with active or inactive IBD” (89 %), “risks of nutrition deficiencies” (83 %), and “nutritional value” (80 %). However, only 8 % to 16 % were satisfied with the amount of information they had received [16].

Food is more than merely nutrition. Food and eating are an integral part of daily life, activities, and well-being. Almost all social events such as holidays, gatherings with friends, parties, weddings, and business meetings revolve around food [2]. This means that any diet recommendation can have an effect on the life of a person with IBD [12]. For example, nearly half of the patients in a French survey reported that the disease negatively affected their enjoyment of food, and 22 % preferred not to eat food outside their homes at all. These experiences and strategies frequently result in feelings of depression and have negative effects on the patient’s social life [12].

While many of these studies are based on standardized questionnaires, there are only a few studies that collected and analyzed data with a qualitative design. In a Canadian study that was published in several parts [5, 17, 18], for example, researchers interviewed eight persons with IBD or irritable bowel syndrome (IBS) about their experiences with the condition. All participants reported that their lives were affected or even controlled by their bowels to some extent [18], and most of them had a strong need for individualized treatment strategies. Such strategies are not commonly provided in current healthcare settings [5]. However, the Canadian study was only based on interviews with young women of between 18 and 23 years, only three of whom had CD and/or UC, the remaining five had IBS [5, 17, 18]. Moreover, the interviews only focused on previously selected issues, such as adverse behaviors, support, and control [5, 18]. Another study with a very narrow scope concentrated on the way patients tried to “cope with their condition centered around food consumption” [17].

These studies failed to report on how the patients live day to day with the limitations in food and nutrition, how these limitations affect their social life and activities, how people try to cope with the resulting difficulties and barriers, and which problems are the most important for them. Making use of the rich interview material from a German website project, we aim to understand from a patient perspective the significance of food, eating, and nutrition, including the role of food in the social life of patients with IBD.

## Methods

### Context and setting

This study is based on full-length interviews conducted for a German website project. Excerpts from these interviews provide the material for the website (www.krankheitserfahrungen.de) and are presented as video, audio, and text interview sequences to display the experiences of people suffering from chronic conditions. To date, the website comprises six different conditions (modules): diabetes, chronic pain, epilepsy, two cancer modules, and IBD. Additional modules are being planned. The project is part of “DIPEx International” (http://www.dipexinternational.org). The main goal of the DIPEx projects is to give patients a platform in which to learn from other patients and to have access to free information that is not provided by medical experts or dubious internet sources. The website can also be used in medical education [19].

### Data collection

As for all modules on www.krankheitserfahrungen.de, we conducted open-end narrative interviews for the IBD module. Interviews took place from September 2011 to June 2012. Maximum-variation sampling was used to include a broad range of experiences and a variety of different factors that might influence people’s experiences [20]. The sample included men and women of different age groups and social and ethnic backgrounds from across Germany.

Furthermore, we interviewed people with different IBD types, disease activities, and prior surgeries (including colectomy, ileostomy, and colostomy). Participants were found through family physicians, support groups, newspaper advertisements, and several online patient forums.

Participants were asked to tell their story from when they first suspected problems with their health. They were encouraged to use their own words and dwell on their thematic preferences, with as little interruption from the interviewer as possible. At the end of the narrative, particular problems mentioned during the narrative were explored in more detail.

Our goal was to collect data that covered the whole range of illness experiences of people with IBD. We therefore used theoretical sampling based on grounded theory methodology [21]. In the first half of the interviews we included men and women of different ages from large and small cities as well as from rural areas in various parts of Germany. We used coding techniques from grounded theory to identify the most important topics and experiences and developed theoretical categories for analysis. Our first results were presented to several external experts (advisory board) in the field of IBD. Because we did not have a predetermined set of categories and themes, we discussed our current sampling and, with the help of the advisory board, identified persons and topics that were not represented in the sample, but were important to represent a broad spectrum of experiences. For example, we became aware that we had interviewed only a few people with mild cases of illness. During the second stage of the study, we therefore focused on patients and experiences that seemed underrepresented (for instance, people who had not experienced much discomfort after the diagnosis) until theoretical saturation was reached; in other words, until no additional new topics and experiences were likely to be found in new interviews [21].

The interviews lasted 30 to 180 min and were digitally recorded (video or audio). Interviews were transcribed verbatim, cross-checked for accuracy by one of the authors (AP), and sent to the interviewees for review and verification. At this stage, the participants were asked to approve the content of the interview and to sign a copyright form giving consent for the interview to be used on the website and for scientific research.

### Analysis

We decided to use a qualitative approach to generate new theories from data according to the topics that were most important to our participants. We used grounded theory, a very common and well-recognized systematic methodology in the social sciences, because this method allows exploring social relationships and the behavior of individuals and groups [21].

This qualitative approach involves moving back and forth in the data to discover common patterns and topics. Furthermore, in keeping with the methodology, we analyzed data while still conducting interviews [21]. We used the qualitative data-indexing software ATLAS.ti to allocate data from each interview to codes that reflected emerging themes; this is based on the method of constant comparison [21]. As the grounded theory approach suggests, the coding process included naming, explanation, and discussion of various concepts of the research area. To do this, two members of the research team (AP and SR) read and re-read the first seven interviews and constructed a coding frame independently of one another (initial coding), using the open coding method that allows interpretations that are close to the interviewees’ formulations [21]. The framework was then compared and was found to be a close match. It was slightly modified and applied to all remaining interviews. This also involved coding newly emerging topics and adding them to the existing framework, which grew in size and complexity and became more abstract in the process. Theoretical memos were used to document the process of how particular codes and themes were derived. The subsequent analysis concentrated on finding overall connections in the interviews. Similar codes were grouped into categories to identify the main themes by framing differentiated concepts (categories). During the interview analysis, we decided to concentrate on the topic of the “significance of food for the participants” because it was the most common topic and was mentioned in almost every interview.

The next step of the analysis was to condense the coded material onto one sheet of paper (OSOP). This is an analysis technique that involves reading through each section of data in turn and noting on a single sheet of paper all the different issues that are raised by the coded extracts, along with the relevant respondent identification [22]. With this method we identified patterns and themes common to all the interviews. The goal was to confirm that the codes and categories accurately represent interview responses and to explore how the concepts and categories are related [23]. In additional team discussions we grouped these aspects into central categories. The method helps to pinpoint the topics and sheds light on how they are connected [22].

All authors of this study regularly reviewed and refined the coding frame and interpretation of the results in several team-meetings to ensure that we did not introduce researcher bias. The identified main categories are the basis for the results section.

### Ethics

The German project, including data analysis for scientific purposes, was approved by the local ethics committee (No. 17/1/07).

## Results

We interviewed 44 persons, two of which ceased to participate for personal reasons. The average age was 42 (median); 54 % of the interviewees were female. Twenty-five of the participants had CD, 15 suffered from UC, and 2 from indeterminate colitis (UC/CD). Additional demographic data are listed in Table 1. Most people were interviewed in their homes, the others came to the department.

**Table 1:** Demographics and patient characteristics

Participant	Diagnosis	Gender	Ranges of age
1	CD	F	Young adult
2	CD	M	Middle-aged adult
3	CD	F	Young adult
4	UC	F	Middle-aged adult
5	CD	F	Young adult
6	UC	F	Middle-aged adult
7	UC	M	Young adult
8	UC/CD	F	Young adult
9	CD	F	Middle-aged adult
10	CD	F	Older adult
11	CD	M	Middle-aged adult
12	UC	M	Young adult
13	UC	M	Older adult
14	CD	F	Young adult
15	UC	F	Young adult
16	UC/CD	M	Young adult
17	UC	M	Young adult
18	UC	M	Middle-aged adult
19	CD	M	Young adult
20	CD	F	Young adult
21	UC	F	Young adult
22	UC	M	Middle-aged adult
23	CD	M	Older adult
24	CD	F	Middle-aged adult
25	UC	M	Middle-aged adult
26	UC	M	Middle-aged adult
27	CD	M	Older adult
28	CD	F	Middle-aged adult
29	UC	F	Young adult
30	CD	F	Older adult
31	CD	F	Middle-aged adult
32	CD	M	Older adult
33	UC	M	Older adult
34	UC	F	Middle-aged adult
35	CD	F	Young adult
36	CD	M	Young adult
37	CD	M	Older adult
38	CD	F	Middle-aged adult
39	CD	F	Middle-aged adult
40	CD	M	Middle-aged adult
41	CD	F	Young adult
42	CD	F	Young adult

*CD* crohn’s disease, *UC* ulcerative colitis, *UC/CD* indeterminate colitis, *M* male, *F* female, Ranges of age: young adults = 17–35, middle-aged adults = 36–55, older adults = 56–79

We chose “significance of food” as the main topic for deeper exploration because almost every participant talked extensively about this, often in a very emotional and touching way. Four interrelated categories emerged from our analysis of eating experiences: managing uncertainty; eating: between craving and aversion; being different; and professional help as a further source of uncertainty. In the following, we describe these categories in detail and illustrate them with meaningful quotes.

### Managing uncertainty

Because eating is often complicated, many of our participants reported that they eat only what they tolerate well. They described the following foods as easily digestible: dry bread, biscuits, steamed vegetables, bananas, and products from their own garden.

*“I’ve never been a big eater, and sometimes it happens that I don’t want to eat anything. Then I just nibble on a bit of dry bread, and that’s it. It always helps me quite well. Even during the strongest acute flare, when I couldn’t eat anything.” (ID 21, female, 24, CD)*

There are many food products which, in the opinion of the interviewees, might lead to more symptoms or even start an acute flare and therefore need to be avoided. Especially the following products were “red flags” in the view of some but not all interviewees: oily and spicy food in general, chocolate, and food causing flatulence, such as cabbage, onions, legumes, whole grains, and fiber-rich food products.

*“There are many things, such as cabbage and beans and even lentils, which you should eat as little as possible or should not eat at all.” (ID 14, male, 72, UC)*

*“I cannot tolerate mushrooms and curry or any extremely spicy or oily food.” (ID 31, female, 17, UC)*

On the other hand, some participants did not have any trouble with food at all.

*“About the food, there are actually no abnormalities with me. I have noticed, if I eat something greasy or something else that doesn’t suit me, then my stool gets more liquid, but if I then eat an apple or a salad then everything will be fine, just how it’s supposed to be” (ID 19, male, 18, CD)*

*“No, I can actually drink and eat what I want. I can tolerate everything, I have no restrictions. (…) Spicy dishes are not a problem.” (ID 36, male, 28, CD)*

During a remission some of the interviewees tried to eat dishes or food that they normally did not tolerate very well.

*“Things that I can tolerate when I’m feeling good are for example sweet peppers (…), but only if I am feeling OK. But then I still have trouble sometimes.” (ID 7, female, 40, UC)*

Lactose intolerance was another common theme. Many interviewees noted that they had trouble in tolerating dairy products. Some began to consume lactose-free products and actually felt better.

*“And lactose I have some problems with. Nothing serious, not a real lactose intolerance (…) but I feel better using lactose-free milk.” (ID 31, female, 17, UC)*

Some interview partners reported food experiences that were rather unusual and often stressful. For instance, they sometimes ate french fries with gyros or cheeseburgers from a fast-food chain even during an acute flare and did not experience any discomforts. One interviewee explained that her body “told” her what she would tolerate by a craving for certain food products.

*“It’s actually funny, it works quite well, my body has a good feeling for what I can or cannot tolerate. So there are things like I’m actually eating a lot of vegetables and salad, but there are phases during which things I always totally liked, I’m now disgusted with, and then I don’t eat them, of course, any longer (…) the feeling can be wrong sometimes, but that’s really quite rare.” (ID 8, female, 31, UC/CD)*

*“Well, if I say to myself: ‘I would like some nice gyros with french fries, and coleslaw, which you should not eat according to every diet recommendations brochures. If I have this craving, then I tolerate it well, it’s actually pretty funny.’” (ID 22, male, 54, UC)*

*“What never was a problem at all is the cheeseburger from (fast-food chain). This is absolutely crazy, it doesn’t matter how bad I’m feeling, cheeseburger is never a problem.” (ID 4, female, 48, UC)*

One person complained about strong frustration because he had done everything ‘correctly’ by restricting himself and by only eating certain dishes and foods, but he still experienced many discomforts.

*“Initially I was upset because I thought, “What have you done wrong this time?” Because after all the diet and what you shall eat and shall not eat, so you had a strict diet and you’ve done nothing wrong, you have been living healthy in the past weeks. You haven’t had any alcohol and yet that [means: the acute flare].” (ID 26, male, 42, UC)*

In the end, almost everybody discovered that there was no such thing as a universal and effective diet for people with IBD. Some of the patients felt that while in remission, they could eat almost anything without any problems, whereas during the times of an acute flare, next to nothing was tolerable. It usually takes a long time to find out compatible and incompatible foods, which often is a very frustrating learning process.

*“It is different for everybody, so there is, I think, in ulcerative (colitis) no special diet that is good or bad. But during the good phase, everyone can tolerate something different well or not.” (ID 5, female, 48, UC)*

*“There are days when you can’t eat anything at all.” (ID 21, female, 24, CD)*

Almost all of our participants told us that they had to find out for themselves which food products they could still tolerate. This was also the advice one interviewee received from his gastroenterologist.

*“You actually have to try out everything for yourself. There are guidelines from the doctors, but basically you have to try out everything for yourself, what you can tolerate and what is not so good or what not at all. Because it is just very different.” (ID 14, male, 72, UC)*

*“Yes, I came up with my own diet. So there are still a few things that I cannot tolerate, or I would not risk, I just no longer try.” (ID 29, female, 45, CD)*

*“And then my gastroenterologist said to me: “Basically,” he said, “you need to try out everything, you have to find out what is best. If you crave something, you should eat it.” (ID 23, male, 54, UC)*

One person proudly reported about a self-developed “food traffic-light system”.

*“I know exactly what I can eat (…). I’m using a kind of traffic-light system. Red are things I never eat, yellow what I can eat when I’m feeling fine and green I can always eat. There is not very much green, though.” (ID 4, female, 48, UC)*

As a result of the high uncertainty connected with the question of which foods to eat, many patients tended to reduce their nutrition during the times of an acute flare. They only consumed foods that they knew they tolerated well, even if it meant eating the same foods for several months.

*“And then we (his doctor and himself) found out what I do tolerate, so really at the end I have eaten in the morning and evening: salami, bread, and an egg, and that was it, actually, with pâté. And that was all I ate the whole time. The whole seven months. It was always the same. I could not stand it quite soon. But everything else I was unable to eat.” (ID 35, male, 57, UC)*

*“There was a time when I really only lived on bananas and cottage cheese (…).” (ID 29, female, 45, CD)*

### Eating: between craving and aversion

Many interviewees told us about restrictions in connection with their diet that were necessary to minimize their discomfort. Many of them found it very difficult to abstain from certain products; they still craved some particular food very much.

*“First you starve, because you are on an IV.”And then you start to loose it because you think about Nutella. And you want to eat a piece of bread or something because you starve and languish for weeks. (…) And then I eventually begged the doctor: Please, cut the crap out of me, I would like to go with my daughter for an ice cream. Because I lost so much weight and you get so crazy. (…) You get mad from hunger, although you are not really hungry, but you want to eat something again. This idea of food (…).” (ID 33, female, 48, CD)*

*“If you spend several years in a hospital because of an acute flare, you start dreaming to be able to eat a mountain of french fries. Although I am not really fond of french fries or fried things. (…) Or just wish that you could eat a mountain of ice cream and that you could actually eat because you can tolerate it.” (ID 29, female, 45, CD)*

The craving was sometimes so intense that some patients ate too much and even had to be hospitalized.

*“And I said to my husband “I’m still hungry” and he said “let’s put a pizza in the oven” and then he came up with the great idea to eat four toasts with a lot Nutella at 9 p.m. and I was feeling queasy (…) and on Sunday I had to go to the emergency room.” (ID 1, female, 30, CD)*

Patients mentioned one particular problem very frequently: even though they knew that they could not eat something, they sometimes ate it anyway. This meant that some participants consumed something they craved, even though they knew they would experience some problems some hours later or maybe the next day.

*“Yes, when I crave something (french fires) (…). But I knew: “You have to ‘pay the price’”, but I did not care. I was willing to risk the consequences.” (ID 29, female, 45, CD)*

*“I like roast pork very much, but when it’s dry, you can forget it. There has to be a little fat. Or nice pork knuckles. So I treat myself twice a year. But I know exactly, the next two days I will have some problems. But I can’t give it up completely. I don’t do it every day.” (ID 24, male, 60, CD)*

Many reported that it was sometimes very distressing to eat anything at all. One interview partner managed to eat nothing during the day, with the aim to control the complaints during the working hours.

*“So for me, it’s normal that I don’t eat all day and only in the evening. For me it is not bad at all, though.” (ID 9, female, 31, UC)*

Consequently, some interviewees developed fears of, or even aversion towards, eating and drinking. One person tried to abstain from eating and drinking as much as possible to avoid problems. This strategy eventually led to huge weight loss and to feeling tired all the time.

*“Problems with my weight, because you are afraid to eat. Because I know exactly that every time I eat something, I feel sick and have to practically run to the toilet and have diarrhea again. (…) I try to eat only the essentials. Drinking is exactly the same. I hardly drink anything (…).” (ID 34, male, 58, CD)*

Many found it difficult to maintain a desired weight because there were only so few foods they tolerated. They clearly saw the risk of malnutrition.

*“And of course, the whole thing makes it all very difficult, to maintain a normal weight. (…) And she [nutritionist] really helps me to find a way to get sufficient nutrients despite all and to keep the weight and maybe not to slip into a situation of malnutrition. This is of course a great danger here, when you suddenly no longer tolerate so many things.” (ID 15, female, 35, CD)*

### Being different

Many participants described difficulties when attending ceremonial occasions such as weddings, birthdays, or when going out to a restaurant.

*“Yes, the very fact that I cannot eat everything in a restaurant makes a visit quite difficult for me. I mean, it is possible, but it is difficult.” (ID 1, female, 30, CD)*

*“Yes, last year was just complicated; in May, my son got married. There was a very big celebration and everything and I didn’t feel good and most of the food I could only look at. (…) If you can only eat a small amount of diet food, but everything else is impossible, and then you realize, there are just some things you cannot participate in anymore.” (ID 37, male, 60, CD)*

At social events, people with IBD had to pay special attention to the food they eat because they could not always find out how the dishes were prepared and whether there was some ingredient they did not tolerate. Furthermore, they often had to explain to other guests why they did not wish to eat particular food products. This sometimes was very awkward and caused to people to go out less frequently or no longer at all.

*“Yes, the issue with food is difficult. Even more so because even if I feel good, I have to reveal myself to others sometimes. You always have to explain. Recently, while at a barbeque, I do not know why, but I said: “No, I cannot eat this.” There was, I think, something like guacamole, and I say, “Yes, there are onions in it,” and one of the BBQ guests asks “Why? Onions are good. Why don’t you eat onions?”, but I did not wish to explain.” (ID 29, female, 45, CD)*

A few women, but no men, reported being considered anorexic because they refrained from eating or frequently had to go to the bathroom during a meal. Some of them felt discriminated.

*“And I remember that before that I was diagnosed, there was a rumor in my family that I had bulimia or anorexia. Because I had to go to the restroom after the meal. Even If it was just to pee. But nevertheless, there was already a comment: ‘Oh, really just to use the toilet?’” (ID 44, female, 28, CD)*

Many patients felt stressed because they had to cook different dishes for themselves and their families at home.

*“And so I have to cook two meals at home because my husband likes it solid, with a lot of cream sauce, and I cannot (eat it).” (ID 1, female, 30, CD )*

Some expressed concerns about travelling because of the lack of opportunities for cooking and the restrictions in preparing food.

*“We flew to Spain in February and I was a little bit concerned. Because of another country with different food. We thought if things get really bad, then we are going back. And everything was great, everything was beautiful, but after about three or four days I got diarrhea and it went on for days and I thought we would have to go home again (…).” (ID 1, female, 30, CD)*

### Professional help as a further source of uncertainty

Participants who sought qualified diet counselling went to doctors, dietitians, alternative practitioners, or found some helpful instructions in the numerous brochures for IBD patients. Some complained about a lack of support or different and confusing guidelines.

*“The doctor gave me some brochures like “cooking with ulcerative colitis” and “diet recommendations”. (…) And it was helpful in the beginning.” (ID 23, male, 54, UC)*

*“She (alternative practitioner) helped me, we discovered things I couldn’t tolerate, and this means a lot.” (ID 37, male, 60, CD)*

*“Then the doctor came in and said: “You are eating. What are you doing?” This was a surprise for me because the others (other doctors) in the rehab hospital always tried to persuade me to eat more.” (ID 7, female, 40, UC)*

Some patients told us that their doctor or nutritionist had recommended eating gluten-free food, sometimes even without proper tests. They felt left alone with the consequences of this information.

*“So then he (the doctor) made a test: ‘Yes, positive’, he said: ‘Yes, well, seems you have a gluten intolerance, you should read up on it online and live accordingly. (…) So then I got the first nervous breakdown. (…) Because it is very dramatic, because you cannot shop for groceries like you used to and you cannot eat normally.” (ID 42, male, 42, CD)*

*“They (the doctors) thought for a long time that I had gluten intolerance and should follow this diet. I remember, it was really hard for me as a child. (…) But when I learned that this wasn’t true, I remember, we had been waiting for the test results on the phone and I had a roll in my hand. As soon as my mom said, you do not have it, I ate this bread so gratefully. That was nice. That was a nice experience.” (ID 43, female, 28, CD)*

A very disturbing experience for some interviewees was that hospital food caused even more stomach problems.

*“And the food (in the hospital) was an extremely greasy soup you actually shouldn’t give someone who just had a stomach operation, I didn’t understand, I had real strong spasms after eating and was feeling really bad.” (ID 3, female, 31, CD)*

*“And then they (nurses) brought me something and I knew I could not eat it. I had specified it earlier, but it was still on the tray. I looked at this tray and just started to cry and was upset. I thought it was just so awful that I could not eat anything (…).” (ID 29, female, 45, CD)*

## Discussion

The aim of this study was to better understand how people with IBD experience food and eating. Nearly all of our participants felt strongly restricted in their eating behavior and the related social activities. Even after many years of living with the illness, food was still a source of uncertainty. Apart from many positive instances, several patients with IBD did not feel supported by their doctors or the health system in diet counselling.

Similar to other studies, participants in our research felt very uncertain about specific food products, for example, milk, sugar, and fibers [2, 12, 17, 24, 25]. Some of them told us about particular foods that they could not tolerate at all, while others had no problems with these same products. But even when a satisfying and effective diet was achieved after painstaking efforts, the tolerance for particular foods might still change over time, resulting in a lifelong struggle and learning process, as Fletcher and Schneider [17] phrased this. Many respondents found it especially frustrating that even when adhering to a strict diet, they were still suffering from severe diarrhea, bleeding, or acute flare-ups. Similar to other studies, our results confirm that determining the “correct” food is a long process fraught with dissatisfaction and uncertainty [2, 8, 12, 17]. However, in contrast to other studies, it was possible for at least some patients in our study to eat everything and enjoy it without any negative consequences.

Eating not only has a functional significance, it also is a very emotional experience. Compared with other studies [13, 14, 17], our results clearly demonstrate how painful food and eating (or not eating, in some cases) can be. Some participants told us about being preoccupied with thoughts about eating most of the day and even in their sleep. In this context, the craving described by some of the interviewees is reminiscent of physical or psychological addiction. For them, food was no longer a joy and a pleasure, but became an agonizing problem. Some participants even tried to eat as little as possible to avoid any possible problems, typically resulting in weight loss. Others would, on occasion, knowingly eat certain foods that lead to problems [26]. A particular challenge for some individuals was being forced to eat only lactose-free or gluten-free food.

Although the patients’ perception of food is clearly very important, the influence it has on their eating behavior or the relation to social activities has been studied only rarely. Only a few studies focused on dietary beliefs and behavior of people with IBD, and the reported results are limited [13, 14]. Similar to the patients in these studies, our participants reported difficulties in social situations that were caused by their complaints.

At the same time, we became aware of some experiences and events that have not been reported to date. For example, our interviewees described social events or journeys that became upsetting and awkward because the patients were concerned they might not be able to eat the provided food, have to explain themselves to other guests, or to listen to some wild speculations about their condition, for instance, anorexia nervosa.

An extreme strategy used by some persons with IBD was to eat or drink almost nothing at all because food always caused pain and discomfort. On the other hand, some interviewees convincingly described surprising and almost unbelievable experiences, such as being able to enjoy even food from fast-food chains or almost anything they wanted by simply “listening” to their bodies. These different experiences and strategies mirror the complex process of dietary change and adopting a new eating behavior. People redefine food choice values, discover new ways of balancing beliefs, and use a trial-and-error approach to revise a food choice script, as we know from food choice models [27].

The patients in our study considered brochures and other material about food helpful during the onset of the disease, as also described in other studies [28, 29]. Some patients in our study, however, complained or were confused about the information received from doctors and dietitians regarding specific dietary strategies for people with IBD. Our findings are similar to those in a recent survey about information needs of patients with long-standing IBD [16] and may be an indication for doctors that their behavior may even increase patient’s uncertainty. This underlines the importance of dietary guidance not only in the first few months after the diagnosis, but also after several years. Dietary guidance is so important because no diet has been proven to have a significant impact [30]. Physicians are often asked for advice, but struggle to give some good recommendations [7]. As a result, many patients make dietary changes without proper professional advice and risk nutritional inadequacies.

There are some implications based on our results that might help persons with IBD and contribute to a better doctor-patient-relationship. It might be helpful, for instance, to prepare patients for the challenge of finding out by themselves which and when certain food products are well or poorly tolerated, even if these products may be in conflict with guidelines and counselors. The patients need to be aware that they will need time, energy, and social support from others to establish new food routines in their life [27]. Especially people who think they have done everything correctly and have strictly followed dietary recommendations need to be made aware that diet may not play a large role in every patient’s symptoms. This will enable them to protect themselves against self-blame and prevent a sense of failure and frustration in case of discomforts. According to our findings, doctors need to establish common ground with their patients. Understanding the patients’ needs is more important than establishing behavior as safe or harmful.

We know from an interview study in a British hospital [31] that patients with IBD expressed the wish that a confident and capable doctor would listen attentively and inquire as to the effects of their illness. The interview situation in our study could be a model for a doctor-patient relation, where patients at least sometimes have the opportunity to tell doctors and other health professionals of their burdensome histories as well as of frustrations with food and eating. This would make them feel less alone with their craving for forbidden food or their unorthodox, sometimes dangerous symptom-avoiding strategies. Doctors should acknowledge the uncertainty that comes with the condition and communicate this to their patients, allowing and encouraging them to talk about their uncertainties. For example, doctors might elicit accounts of patients’ fears and advise them to occasionally eat different food products so that patients can then determine whether they tolerate these after all, because intolerance can change over time.

Future research should concentrate on finding patterns for well-tolerated food from the patient perspective. This could be achieved, for example, by motivating IBD patients to keep long-term dietary diaries. These could then be analyzed. The idea of one interviewee of developing a “food traffic-light system” may be another way to become more active and engaged through individualized diet management. This could be a topic of further research.

It is still unclear whether patient expectations of a specific food product or the product itself are the decisive factor in causing a patient to feel unwell. Given that craving for “forbidden” food and frustration caused by not knowing what to eat are two of the strongest concerns for persons with IBD, it is important to determine whether they might benefit from support groups with an emphasis on food, or from cooking classes in which they can share experiences. Another topic that needs to be investigated is the possible social isolation of the patients, which is a result of their being afraid to declare their condition at public events or being afraid to eat and drink at all, even when alone.

### Strengths and limitations of the study

In contrast to other qualitative research about IBD [5, 13, 18], our samples are larger and more diverse because maximum-variation was an important criterion during the sampling. However, a certain selection bias has to be discussed: As described in the Method section, one important study aim was to publish the interviews or parts of them on the website www.krankheitserfahrungen.de. For ethical reasons, we openly stated this in all our acquisitions, that is, in the advertisements in newspapers, online forums, etc. To know that they would be featured on the Internet might have been an argument for some people to decline. It is possible that these decliners have certain characteristics and experiences in common that are underrepresented in our interview material.

The open-end narrative character of our interviews allowed the participants to address the problems that were import to them. Almost all of the interviewees talked about food and nutrition, which reflects the immense importance of this topic for patients with IBD. In addition to other studies, the open nature of our interviews gave our participants room to describe how they handled uncertainty in everyday life, which unusual situations they experienced, and how important it was for them to behave unreasonably from time to time.

As with any qualitative study, data collection and analysis can be influenced by the researchers’ personal biases and idiosyncrasies, especially during the interview, when selecting the quotes or interpreting the data. We mitigated this by working in an interdisciplinary team that consisted of medical doctors, a psychologist, and sociologists. Similarly to other reports, our attempts to combine universal observations about problems related to food are limited because qualitative studies typically have only a few participants. Eating habits and the social environment of eating are strongly influenced by cultural components. It is possible that our findings cannot be applied to other countries.

## Conclusion

The participants of this study freely reported difficulties and exceptional strategies and experiences in their daily struggle with their illness. A very frequent experience of our interviewees was difficulty in suppressing the craving for and abstaining from certain food products and the failure to do so, despite the consequences. Others described their fears and aversions towards eating in general or reported that they were unable to eat the food provided at social events. Because of this, some of our interviewees began to avoid going out and eating in public, which in turn again increased their social isolation and had a negative effect on their social relationships. Health professionals need to become more involved and not only give advice about food and eating, but also try to find strategies with their patients of how to avoid social isolation because of dietary problems, for example.

## Abbreviations

IBD: Inflammatory bowel diseases
UC: Ulcerative colitis
CD: Crohn’s disease
IBS: Irritable bowel syndrome
OSOP: One sheet of paper
